# Comparative Analysis of the Total Proteome in Nonalcoholic Steatohepatitis: Identification of Potential Biomarkers

**DOI:** 10.1016/j.mcpro.2025.100921

**Published:** 2025-01-31

**Authors:** Eda Ates, Hien Thi My Ong, Seung-Min Yu, Ji-Hoon Kim, Min-Jung Kang

**Affiliations:** 1 Center for Advanced Biomolecular Recognition, Biomedical Research Institute, Korea Institute of Science and Technology, Seoul, Republic of Korea; 2 Division of Bio-Medical Science & Technology, KIST School, University of Science and Technology, Seoul, Republic of Korea; 3 College of Medicine, Seoul National University, Seoul, Republic of Korea

**Keywords:** NAFLD, NASH, biomarker, proteomics, multiple reaction monitoring

## Abstract

Nonalcoholic fatty liver disease is a hepatic condition characterized by excessive fat accumulation in the liver with advanced stage nonalcoholic steatohepatitis (NASH), potentially leading to liver fibrosis, cirrhosis, and cancer. Currently, the identification and classification of NASH require invasive liver biopsy, which has certain limitations. Mass spectrometry-based proteomics can detect crucial proteins and pathways implicated in NASH development and progression. We collected the liver and serum samples from choline-deficient, L-amino acid-defined high-fat diet fed NASH C57BL/6J mice and human serum samples to examine proteomic alterations and identify early biomarkers for NASH diagnosis. In-depth targeted multiple reaction monitoring scanning and immunoblotting assays were used to verify the biomarker candidates from mouse liver and serum samples, and enzyme-linked immunosorbent assay (ELISA) was employed to analyze human serum samples. The multiple reaction monitoring analysis of NASH liver revealed 50 proteins with altered expression (21 upregulated and 29 downregulated) that are involved in biological processes such as detoxification, fibrosis, inflammation, and fatty acid metabolism. Ingenuity pathway analysis identified impaired protein synthesis, cellular stress and defense, cellular processes and communication, and metabolism in NASH mouse liver. Immunoblotting analysis confirmed that the expression of proteins associated with fatty acid metabolism (Aldo B and Fasn) and urea cycle (Arg1, Cps1, and Otc) was altered in the mouse liver and serum. Further analysis on human serum samples using ELISA confirmed the increased expression of multiple proteins, including Aldo B, Asl, and Lgals3, demonstrating values of 0.917, 0.979, and 0.965 of area under the curve in NASH diagnosis. These findings offer valuable insights into the molecular mechanisms of NASH and possible diagnostic biomarkers for early detection.

## Highlights

• MS-based proteomics used to identify key proteins and pathways in NASH development.
• Pathway analysis revealed disruptions in fibrosis, detoxification, and metabolism.
• Human serum analysis showed biomarkers with high diagnostic accuracy up to 0.979 AUC.
• Biomarkers Aldo B, Asl, and Lgals3 offer hope for early NASH diagnosis and treatment.
• A multilevel verification approach enhances the robustness and reliability of NASH findings.

## Graphical Abstract

**Figure:**
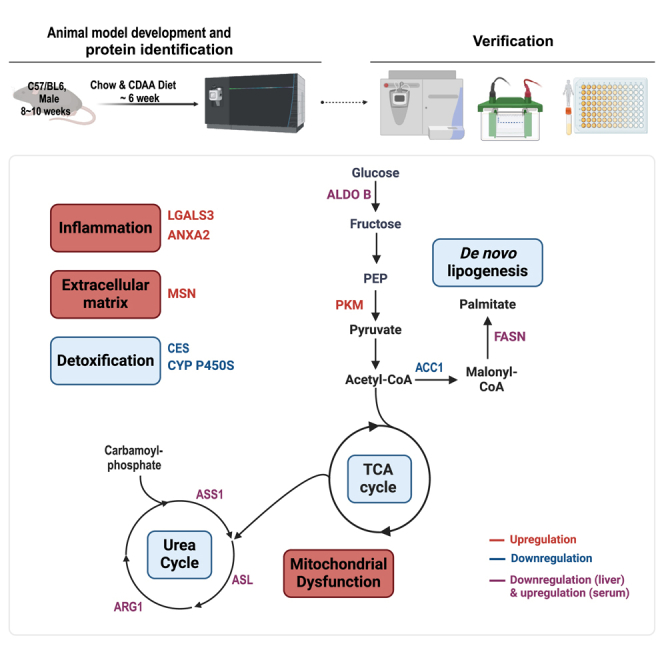

## In Brief

This study examined the molecular mechanisms of nonalcoholic steatohepatitis (NASH) and sought to identify tissue specific early diagnostic biomarkers by analyzing combination proteomics of tissue and serum. Using well-defined mouse models and proteomic analysis, differentially expressed proteins in NASH were identified. Disruptions in various cellular functions, including protein folding, lipid metabolism, and energy production, were observed. Pathways related to inflammation, fibrosis, oxidative stress, and mitochondrial dysfunction were also altered in NASH. The findings were verified in human serum samples, with several potential biomarkers identified for NASH diagnosis.

Nonalcoholic fatty liver disease (NAFLD) constitutes a broad spectrum of liver conditions characterized by excess fat content in the liver, typically exceeding 5% of the liver’s weight. Different studies have reported that NAFLD affects 25% of the global population, particularly those with obesity and diabetes (1). NAFLD ranges from simple steatosis (excessive hepatic triglyceride accumulation) to a harmful form known as nonalcoholic steatohepatitis (NASH). NASH is a complex condition characterized by fat accumulation, hepatocyte ballooning, different levels of fibrosis, and inflammation (2). It is a potentially dangerous condition owing to its ability to lead to other severe liver diseases. Ten to twenty-five percent of patients with NASH could progress to cirrhosis and/or hepatocellular carcinoma, potentially leading to death (3). Therefore, the diagnosis of NASH in its early stages is critical for controlling and preventing the mortality associated with this extremely prevalent disease.

The diagnosis and staging of NASH and fibrosis have traditionally been assessed using the gold standard liver biopsy method. However, this invasive procedure has several limitations that hinder its widespread use. Liver biopsies are prone to sample bias and sampling variability because they only provide a snapshot of the liver tissue at a specific location. Furthermore, in certain individuals, liver biopsies can result in complications, such as bleeding, and cannot be repeated for tracking disease progression over time (4). Due to the challenges associated with this invasive technique, there is a need for alternative diagnostic tools that are reliable, noninvasive, and accurate.

Omics technologies have undergone significant advancements in recent years. Proteomic analysis is a valuable tool for unraveling the complex molecular pathways involved in NASH progression. By analyzing the proteome, key proteins and pathways that contribute to NASH development and progression can be identified, along with novel biomarkers. Mass spectrometry (MS)-based proteomic analysis could potentially be a solution for developing methods to analyze big data for the detection and identification of novel early biomarkers. Diagnostic biomarkers can be considered objective and impartial indicators that can differentiate between the onset of an illness and a healthy state. They also assist in categorizing whether a person suffers from a disease and can help determine the stage and severity of the disease.

Ultimately, it is crucial to explore the molecular mechanisms responsible for NASH progression to enhance patient outcomes through early detection and the development of precise treatments. In the current study, using advanced proteomic techniques, we obtained a NASH mouse model with choline-deficient L-amino acid-defined high fat (CDAA-HF) and analyzed mouse serum, liver samples, and human serum samples to detect an early biomarker for NASH diagnosis. The validation of key protein candidates using various analytical methods further strengthens the credibility of the results. Ultimately, this study has the potential to advance our knowledge of NASH progression and pave the way for the development of novel diagnostic and therapeutic strategies.

## Experimental Procedures

### Experimental Design and Statistical Rationale

To closely mimic NASH in mice, 8–10-week-old male C57BL/6J mice (n = 35) were obtained from Raonbio (Gyeonggi-do, South Korea) and randomly assigned to either the control (chaw-diet) group (n = 2–3) or the CDAA-HF diet NASH group (n = 3–7). The mice (2–4 per cage) were maintained under a standard 12-h light/dark cycle. Throughout the experiment, both the groups had unrestricted access to food and tap water. The body weights of the mice were measured weekly. At predefined weekly intervals (at weeks 6, 14, 16, and 18), the mice were euthanized, and liver specimens and sera were collected for subsequent molecular analyses. The differential protein expressions (DEPs) of week six liver samples were examined for the early biomarker candidates. The candidates were verified in different studies using multiple reaction monitoring (MRM), immunoblotting, and ELISA.

All experiments were performed in biological triplicates. The data are expressed as the mean ± standard deviation. Comparisons among multiple groups were made by one-way ANOVA, and the comparison of two groups was performed using Student’s *t* test. Differences were considered significant at *p* value < 0.05. All graphs were generated using the GraphPad Prism software (version 11.0, MA, USA, www.graphpad.com), and graphical abstract was created with Biorender.com.

### Protein Extraction and Immunoglobulin G and Albumin Depletion

Liver tissues were homogenized using BioMasher III (Nippi Inc) and incubated in radioimmunoprecipitation assay buffer (Cell Signaling Technology) with protease and phosphatase inhibitor (Cell Signaling Technology) for 20 min at 4 °C. To ensure complete lysis, the cells were sonicated for 30 s. The lysate was then centrifuged at 14,000*g* for 10 min at 4 °C. The resulting supernatant was transferred into a fresh tube, and protein concentration was quantified using the Pierce bicinchoninic acid Protein Assay Kit (Thermo Fisher Scientific) with bovine serum albumin as the standard, following the manufacturer’s protocol. The samples were then stored at −80 °C until further analysis.

Mouse serum samples were subjected to immunoglobulin G and albumin depletion prior to further analysis. Proteome Purify 2 Mouse Serum Protein Immunodepletion Resin (Bio-Techne, R&D Systems) was used for depletion, according to the manufacturer’s protocol. The serum samples (10 μl) were incubated at room temperature (RT) in 1 ml of immunodepletion resin for 1 h in a rotary shaker. After the incubation period, equal volumes of the serum and immunoresin mixture were loaded into a 0.22-μm Ultrafree Centrifugal Filter (Millipore), followed by centrifugation for 2 min at 1500*g*. The flow-through was collected and stored at −20 °C until further analysis.

### In-Solution Digestion

The extracted proteins were reduced using DTT (Sigma-Aldrich) at 56 °C for 40 min. The samples were then alkylated with iodoacetamide (Sigma-Aldrich) in the dark at RT for 30 min. Iodoacetamide activity was reduced using DTT in the dark at RT for 10 min. The samples were digested with trypsin (MS Grade, Sigma-Aldrich) at a 1:30 enzyme/protein concentration ratio for 24 h at 37 °C. The digestion was stopped by reducing pH with 5 μl of formic acid (FA, Thermo Fisher Scientific). The samples were completely dried using an Alpha 1 to 2 LD plus vacuum freeze-dryer (Martin Christ).

Pierce Peptide Desalting Spin Columns (Thermo Fisher Scientific) were used for desalting the samples, following the manufacturer’s protocol. Briefly, the spin columns were conditioned by washing the resin twice with 100% acetone and twice with 0.1% TFA (Thermo Fisher Scientific), followed by centrifugation at 5000*g* for 1 min each. The digested and dried peptides were dissolved in 0.1% TFA solution, loaded onto a conditioned column, and centrifuged at 3000*g* for 1 min. The flow-through was discarded, and the resin was washed thrice with 0.1% TFA solution and centrifuged at 3000*g* for 1 min. Finally, the peptides were eluted from the resin by washing the column twice with 0.1% TFA in 50% acetonitrile solution, and the eluate was collected in fresh Eppendorf tubes after centrifugation at 3000*g* for 1 min. The eluate was completely dried using a vacuum freeze dryer until complete drying was achieved. Next, the dried peptides were either stored at −20 °C or resuspended in 150 μl of 0.1% FA for MS analysis.

### Mass Spectrometry

The digested peptides were subjected to nano-liquid chromatography with tandem mass spectrometry (LC-MS/MS) using an Orbitrap Eclipse Tribrid Mass Spectrometer (Thermo Fisher Scientific) coupled with an Ultimate 3000 Rs nano (Thermo Fisher Scientific). The samples were loaded onto the analytical column and separated using reversed-phase chromatography. The separation was performed on a 50-cm column (Acclaim PepMap RSLC C18, Cat. No: ES903) with an inner diameter of 75 μm and packed with 2 μm C18 particles (Thermo Fisher Scientific). The samples were eluted from the nanocolumn with multistep gradients of 5 to 40% solvent B (A: 0.1% FA in water; B: 0.1% FA in acetonitrile) over 102 min at a flow rate of 300 nl/min, with a total run time of 120 min. The MS was operated in positive ionization mode with a nanospray voltage set at 1.90 kV and ion transfer tube temperature at 275 °C. A maximum injection volume of 5 μl was used during data acquisition with partial injection mode. The MS was controlled in a data-dependent mode, which was toggled automatically between MS and MS/MS acquisition. The data-dependent mode was set to include 2 to 4 charge states, excluding undetermined charges, an exclusion duration of 60 s, mass tolerance ppm for low 20, and excluded isotopes. MS/MS data acquisition and processing were performed using Xcalibur software (Thermo Fisher Scientific, www.thermofisher.com).

### Protein Identification and Quantitative Analysis

Proteins were identified using the Proteome Discoverer software (ver. 2.5, Thermo Fisher Scientific, www.thermofisher.com) and mouse (*Mus musculus*) UniProt protein sequence database. The reviewed mouse protein sequences were downloaded from the UniProt protein database on September 13, 2023. The UniProt databases used were as follows: *M. musculus* (sp_canonical, Entry number: 17163*), M. musculus* (tr_canonical, Entry number: 70629), *M. musculus* (sp_incl_isoforms, Entry number: 25524), *M. musculus* (sp_tr_canonical, Entry number: 85010), and *M. musculus* (sp_tr_incl_isoforms, Entry Number: 92954). SEQUEST uses carbamidomethylation of cysteine as a static modification and oxidation of methionine as a dynamic modification in the search for normal peptides. Maximum missed cleavage sites for trypsin, a proteolytic enzyme, were set to two. Precursor and fragment mass tolerances were configured to ±10 ppm and 0.6 Da, respectively, with a precursor mass range of 350 to 5000 Da, and peptide charges were set excluding +1. SEQUEST HT results were filtered using percolator-based scoring to improve the sensitivity and accuracy of peptide identification. False discovery rates, a threshold setting in Proteome Discoverer that indicates a low false positive proportion for identifications, were established to minimize false positives. False discovery rates was set to high (1%) for protein and peptide levels to ensure that the majority of identified proteins and peptides are true positives, thereby increasing the reliability of the results.

### Biological Pathway Analysis

The proteomic dataset, which included UniProt identifiers and fold changes of total identified proteins, was subjected to core analysis using Ingenuity Pathway Analysis (IPA) (Ingenuity Systems). Protein interactions, pathways, upstream regulatory analyses, and functional networks were generated using IPA. The protein matched with the submitted dataset in the Ingenuity Knowledge Base generated molecular networks based on biological and molecular functions, including canonical pathways, upstream regulatory analysis, and disease-based functions, for discovering biomarkers. Core analysis was performed with the settings of indirect and direct relationships between molecules based on experimentally observed data, considering data sources from mouse databases in the Ingenuity Knowledge Base.

### MRM Scanning

MRM was performed for targeted quantification of specific peptides. MRM analysis was conducted by optimizing the MRM transitions for the selected peptides, and the samples were analyzed using scheduled MRM acquisitions. The digested peptides from the liver and serum proteins were analyzed using an Orbitrap Velos Pro Mass Spectrometer (Thermo Fisher Scientific), coupled with an Ultimate 3000 UHPLC system (Thermo Fisher Scientific). Peptides were loaded onto the analytical column and separated by reversed-phase chromatography using a 50 mm column (Accucore C18 HPLC Columns, Cat. No: 17126–052130) with an inner diameter of 2.1 mm, packed with 2.6 μm C18 particles (Thermo Fisher Scientific). The samples were eluted from the C18 column with multistep gradients of 0 to 40% solvent B (A: 0.1% FA in water; B: 0.1% FA in acetonitrile) over 11 min, followed by 40 to 100% for 11 min, 100% for 2 min, and 0% for 5 min, with a flow rate of 250 μl/min and a total run time of 30 min. The MS was operated in positive ionization mode along with electrospray ionization, with the source’s spray voltage set to 4.0 kV and the heater temperature to 275 °C. A maximum injection volume of 20 μl was used during data acquisition with partial injection mode.

### Development and Analytical Validation Targeted MS Assays and Measurements

The MRM assay was developed on Orbitrap Velos Pro MS instrument following guidelines for Tier 3 assays, with data acquisition and processing conducted using the Xcalibur software. The peptides for the MRM analysis selected from the discovery experiment are in the abovementioned Mass Spectrometry section. Initially, peptides were chosen based on their uniqueness to the relevant proteins; in cases where no suitable unique peptides were present, those with high abundance and lowest number of shared proteins were prioritized. For those, the fragmentation sequences were selected from 7 to 30 amino acids for the ionization and fragmentation efficiency. Thus, for each candidate peptide, a detailed examination of the fragmentation patterns derived from the MS/MS spectra was examined. Finally, precursor ions were selected based on their ability to produce distinct and abundant product ions, which are essential for accurate quantification in the MRM workflow. As for the product ions, the highest abundant peak was selected. Any coeluting peptides were placed in different methods to avoid any interference and overlapping during the detection. All peptides are listed in Table S-1 along with all other information including fragment information, coverage, and peptide number.

Label-free peptide quantification was performed using Thermo Xcalibur Qual Browser (Thermo Fisher Scientific). To confirm peptide identity, full-scan analysis of MS2 spectrum of the peptide was conducted to ensure that the retention time and fragmentation patterns matched those of the target proteins. Following confirmation, all the peak areas used for concentration calculation were inspected manually to avoid possible interference in the mass spectra.

### Immunoblotting Assays

Equal amounts of protein were denatured, and 10% SDS PAGE was performed. Protein samples were electrophoretically transferred onto nitrocellulose membranes (Bio-Rad). Nonspecific protein-binding sites were blocked with Tris-buffered saline containing 0.1% Tween-20 and 5% nonfat dried milk for 1 h at RT. After blocking, the membrane was probed with primary antibodies against fatty acid synthase (Fasn), fatty acid binding protein 1 (Fabp1) (Cell Signaling Technology), fructose-bisphosphate aldolase B (Aldo B), ornithine transcarbamylase (Otc), carbamoyl-phosphate synthase 1 (Cps1), and Arginase 1 (Arg1). (Thermo Fisher Scientific). After washing with Tris-buffered saline containing 0.1% Tween-20, the membranes were incubated with horseradish peroxidase-conjugated secondary antibodies at RT for 1 h. The membranes were detected using enhanced chemiluminescence (Santa Cruz Biotechnology) and exposed to Ez-Capture MG (Atto). Protein expression levels were quantitatively analyzed using ImageJ software (version 1.53 t, U.S. National Institute of Health, https://imagej.net/ij/). Due to the structural differences between human and mouse proteins, immunoblotting assays were performed only on mouse serum samples.

### RNA Isolation and RT-PCR

To evaluate the expression levels of the liver fibrosis and inflammation markers, total RNA from mouse liver tissues at designated time points was obtained using TRIzol Reagent (Life Technologies), isolated by chloroform phase separation, and precipitated with isopropanol and ethanol. Complementary DNA synthesis was performed using ReverTraAce (Toyobo), following the manufacturer’s instructions. RT PCR was performed using the SYBR Green PCR Master Mix (QIAGEN), following the manufacturer’s instructions in 96-well plates using a qTOWER 3 Real-Time PCR Thermal Cycler (Analytik Jena). Quantitative PCR (qPCR) was conducted using specific primers for the fibrosis markers *Col1a1* and *Acta2*, as well as the inflammation marker *Mcp1* to assess the progression of fibrosis and inflammation in animal model. All primer sequences are listed in Supplemental Table S2. All measurements were performed in triplicate. The relative mRNA levels were calculated using the 2^-ΔΔCt^ method and normalized to those of *Gapdh*.

### Human Cohort

A total of 50 serum samples from healthy individuals and patients with steatohepatitis were obtained from the Biobank of Ajou University Hospital, a member of the Korea Biobank Network. The healthy group consisted of 25 individuals (18 females and 7 males) with no history of liver disease. The mean age and body mass index (BMI) levels are 47.1 and 20.7 for females and 58.1 and 20.7 for male participants, respectively. Liver function tests revealed mean levels of aspartate aminotransferase (AST) at 19.7 and 20.4 U/L and alanine aminotransferase (ALT) at 16.9 and 18.2 U/L for females and males, respectively. None of the participants reported a history of significant alcohol consumption or use of hepatotoxic medications. The steatohepatitis group also comprised 25 individuals (12 females and 13 males) diagnosed based on criteria. In this cohort, the mean age was 56.0 and 38.1 years for females and males, respectively. The average BMI levels are 27.9 for females and 29.2 for male participants, indicating a higher prevalence of obesity when compared to the healthy controls. Liver function tests showed elevated AST and ALT levels, with means of the female and male as follows 69.5 and 78.2 U/L and 109.6 and 153.6 U/L, respectively (Supplemental Table S3). A detailed drug history revealed that most patients were on medications for hepatoprotective agents (Godex, Ursa, Pennel, Legalon, and Oltipraz) and cholesterol-lowering agents (Livalo and Lipitor). Among the steatohepatitis patients assessed, 11 individuals underwent confirmation of their disease stage. Out of these, six patients had liver biopsies performed, and five patients underwent fibroscan assessments to evaluate their liver stiffness and fibrosis. Fibrosis staging of the patients with the confirmation revealed that 36% as stage 1, 36% as stage 2, and 28% as stage 3, indicating a varying degree of liver fibrosis. Inflammatory stages were assessed, with 60% of patients having mild lobular inflammation and 40% of patients with moderate lobular inflammation. Steatosis staging indicated that 20% of patients were classified as stage 1, 40% as stage 2, and 40% as stage 3, reflecting significant fat accumulation in the liver.

Blood samples were collected using a serum separator tube with a clot activator. No additives such as anticoagulants, preservatives, or protease inhibitors were used during the collection process. Samples were maintained at a temperature of 2 to 12 °C and processed within 2 h after collection. The sample was centrifuged once at 2 to 10 °C for 10 to 15 min at a speed of less than 3000 rpm, with no braking applied. After centrifugation, samples were incubated at 2 to 10 °C for 1 to 2 h storing them in cryotubes (1–2 ml) at temperatures ranging from −85 to −60 °C. Upon receipt, the samples were aliquoted and stored at −85 °C until used. No further freeze-thaw cycles were applied to the samples.

### Enzyme-Linked Immunosorbent Assay

Human pyruvate kinase isozymes M1/M2, Fasn, arginase-1, fructose-biphosphate aldolase b, arginosuccinate lyase, annexin A2, and arginosuccinate synthetase were purchased from MyBioSource. Human galectin-3 quantikine ELISA kit was purchased from R&D Systems. Human moesin ELISA kit was purchased from BT Laboratory. Human serum samples were randomly selected to avoid sample bias, and all biochemical parameters of the samples were measured using ELISA, according to the manufacturer’s protocol. Optical signal densities were measured using an Epoch microplate spectrophotometer (BioTek, Agilent Technologies) or a SPECTROstar Nano spectrophotometer (BMG LABTECH) at 450 and 570 nm.

The data were analyzed using the MedCalc Statistical software (version 20.115, Ostend, Belgium, www.medcalc.org) to determine sensitivity, specificity, and criterion, and the area under the curve (AUC) values to generate a receiver operating characteristic curve.

## Results

### Animal Model

**Fig. 1:**
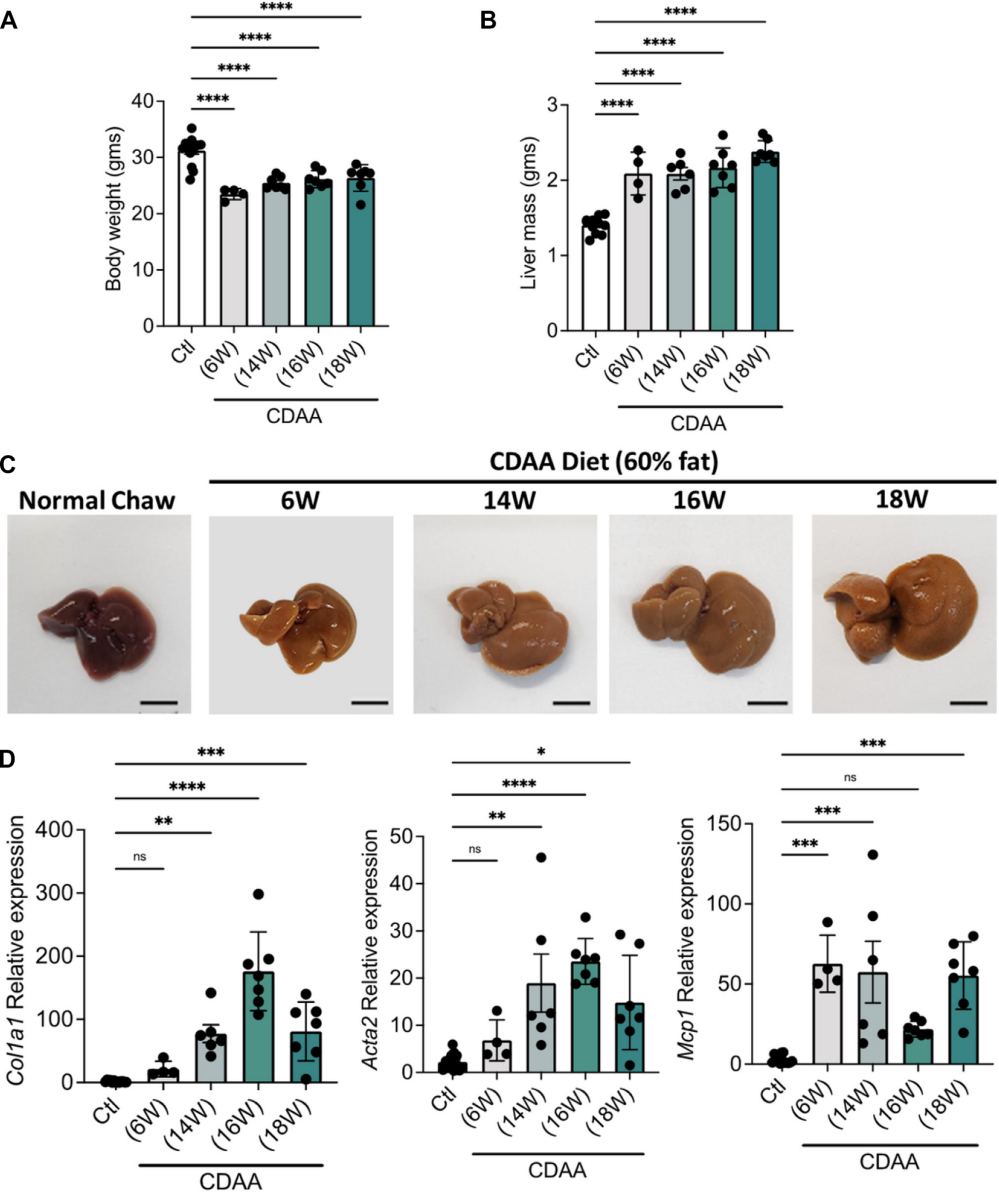
**Comparison of advanced liver disease phenotype and genotype induced by choline-deficient, amino acid-defined (CDAA) diets with high fat (HF) content in C57Bl/6J mice**. Overall changes in body weight (*A*) and liver mass (*B*) after a prolonged CDAA-HF diet. *C*, liver images of CDAA-HF and LF diet-fed mice. *D*, relative expression levels of Col1a1, Acta2, and Mcp1. Statistical analyses were performed using one-way analysis of variance (ANOVA) and the GraphPad Prism software. (∗*p* < 0.05, ∗∗*p* < 0.01 and ∗∗∗*p* < 0.001). ACTA2, alpha-smooth muscle actin; CDAA, choline-deficient L-amino acid; COL1A1, collagen type I alpha 1 chain; MCP1, monocyte chemoattractant protein-1.

The NASH phenotype was induced in C57Bl/6J mice by feeding them a CDAA-HF diet over several weeks. Following treatment, the weight of the mice significantly decreased (Fig. 1*A*), whereas their liver mass significantly increased (Fig. 1*B*). Even at the early stages of CDAA-HF diet treatment, the overall morphology and color of the liver changed, turning brownish (Fig. 1*C*). Fibrosis progression in the NASH mouse model was assessed by measuring the relative gene expression levels of liver fibrosis markers, specifically *Col1a1* and *Acta2*, as well as the inflammation marker *Mcp1*, using RT-qPCR. (Fig. 1*D*). Diet treatment significantly increased the expression of liver fibrosis markers *Col1a1* and *Acta2* starting at week 14 but showed no significant increase at week 6. In contrast, the liver inflammation marker *Mcp1* was significantly elevated starting from week 6. These results suggest that even at the early stages of CDAA-HF diet treatment in mice, there was inflammation in the liver, and starting at week 14, there was a significant increase in liver fibrosis.

### Label-free Quantitative Proteomics Analysis of Mouse Liver Tissue

**Fig. 2:**
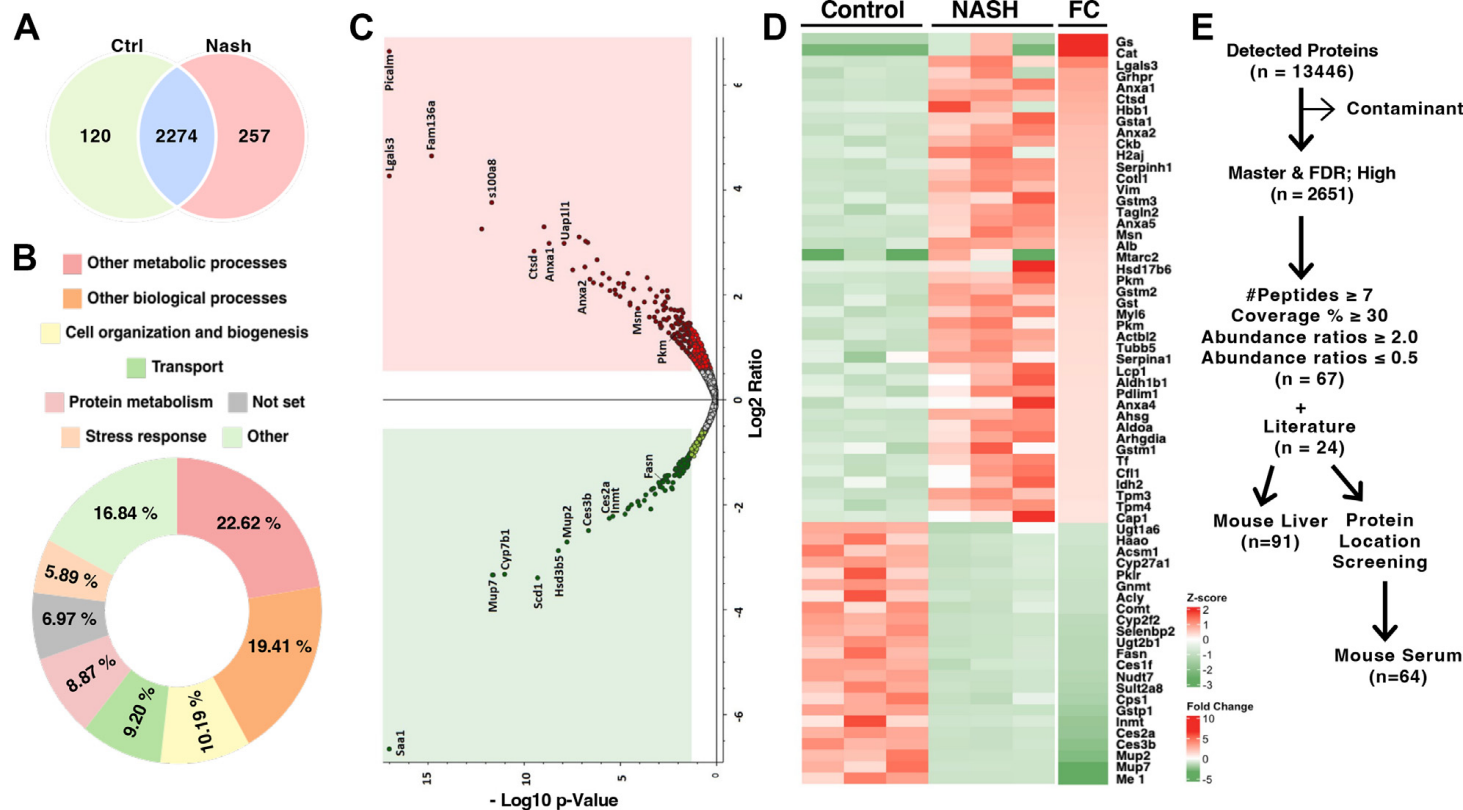
**Differential expression of the NASH proteome**. Differentially expressed proteins between the control and HFD-induced NASH mouse livers. *A*, Venn diagram summarizing the proteins found in the two groups using Proteome Discoverer (Version 2.5) after the removal of contaminants and selection of high-protein FDR-confident master proteins (n = 2651). *B*, Pie diagram representing the percentile distribution of biological processes (n = 2651). *C*, Volcano plot displaying the log2 fold change (*x*-axis) against the *t* test-derived −log 10 statistical *p*-value (*y*-axis) for all proteins differentially expressed among the control and NASH groups (n = 2651). Proteins with significantly decreased levels are shown in *green*, whereas those with significantly increased levels are shown in *red*. *D*, heat map showing the relative abundances of 67 proteins identified across the two groups of mouse liver samples. The color key indicates the relative abundance of each protein (−3–2) across six samples. *E*, protein selection rationale used in the study and multiple reaction monitoring screening. FC, fold change; FDR, false discovery rate; NASH, nonalcoholic steatohepatitis.

The peptide samples obtained from control and NASH liver tissues were analyzed using LC-MS/MS (TIC shown in Supplemental Fig. S1), and the resulting spectra were processed using Proteome Discoverer 2.5 software. More than 13,000 proteins were identified in both groups. After removing the contaminant proteins, master proteins with high false discovery rate confidence were selected (n = 2651), with 2274 proteins common to both groups (Fig. 2*A*). Among the identified proteins, 120 were unique to the control group, and 257 were unique to the NASH group. The identified proteins were primarily associated with biological and metabolic processes, transport, protein metabolism, cellular organization, biogenesis, and other related processes (Fig. 2*B*).

As depicted in the volcano plot (Fig. 2*C*), this approach enabled the identification of differentially expressed proteins, including upregulated inflammatory markers, such as galectin-3 (Lgals3), protein S100-A8 and annexin (Anxa2), and downregulated proteins, such as major urinary protein 7 (Mup7) and cytochrome P450 B1 (Cyp4507b1). Heat map data represent the alterations in NASH samples when compared with those in the control group (Fig. 2*D*). Proteins with significantly decreased levels are shown in green, whereas those with significantly increased levels are shown in red. The heat shock cognate 71 kDa protein (Hspa8) was removed from the heat map because of its high z-score for better map visualization (the heatmap with Hspa8 is presented in Supplemental Fig. S2).

The number of proteins was then reduced to 67 using the following parameters to observe highly altered proteins with statistical significance: peptide number (≥7), coverage (≥30%), and abundance ratios (≤0.5 and ≥2.0). This information is summarized in Table 1. Additionally, 24 proteins that showed a relationship with fibrosis and NASH were added to the list from the literature, and all proteins were used to analyze mouse serum samples using MRM (5, 6, 7, 8, 9, 10, 11, 12). However, further selection was conducted to analyze mouse serum samples by checking the localization of proteins using the UniProt database (https://www.uniprot.org/uniprotkb/, Accessed: April 2024). Proteins located in the cytoplasm, cytosol, and/or secreted proteins were selected for further analysis (Fig. 2*E*).

**Table 1:** List of differentially expressed proteins found in NASH mice after selection

Accession	Gene symbol	Description	# Peptides	Coverage [%]	Abundance ratio
V9GZG5	Gs	submitted name: Glutamine synthetase	12	33	100
Q3UZE7	Cat	Catalase	37	72	100
Q3V471	Lgals3	Galectin-3	7	46	19.314
D6REG4	Grhpr	submitted name: Glyoxylate reductase/hydroxypyruvate reductase	9	47	8.185
Q4FK88	Anxa1	Annexin	16	51	7.954
Q8C243	Ctsd	Peptidase A1 domain-containing protein	12	40	7.156
B1Q450	HBB1	submitted name: Hemoglobin beta chain subunit	12	69	6.371
P13745	Gsta1	Glutathione S-transferase A1	9	35	5.795
P07356	Anxa2	Annexin A2	19	55	4.974
Q04447	Ckb	Creatine kinase B-type	9	35	4.681
Q8R1M2	H2aj	Histone H2A.J	7	41	4.571
Q8BVU9	Serpinh1	Serpin H1	9	34	4.462
Q9CQI6	Cotl1	Coactosin-like protein	7	53	4.284
P20152	Vim	Vimentin	45	79	4.276
P19639	Gstm3	Glutathione S-transferase Mu 3	15	62	4.067
Q9WVA4	Tagln2	Transgelin-2	10	60	3.952
P48036	Anxa5	Annexin A5	31	80	3.613
P26041	Msn	Moesin	25	42	3.358
P07724	Alb	Albumin	67	88	3.002
E0CZH6	Mtarc2	Submitted name: Mitochondrial amidoxime reducing component 2	7	43	2.814
Q9R092	Hsd17b6	17-beta-hydroxysteroid dehydrogenase type 6	12	38	2.719
P52480-2	Pkm	Isoform M1 of pyruvate kinase PKM	24	52	2.693
P15626	Gstm2	Glutathione S-transferase Mu 2	21	74	2.648
Q7M0F4	Gst	Glutathione S-transferase	10	46	2.546
A0A1W2P6F6	Myl6	Submitted name: Myosin, light polypeptide 6, alkali, smooth muscle and nonmuscle	7	58	2.43
P52480	Pkm	Pyruvate kinase PKM	24	55	2.428
P99024	Tubb5	Tubulin beta-5 chain	26	74	2.331
Q8BFZ3	Actbl2	Beta-actin-like protein 2	13	31	2.33
P07758	Serpina1a	Alpha-1-antitrypsin 1–1	14	45	2.302
Q3TJX0	Lcp1	Plastin-2	35	62	2.284
Q9CZS1	Aldh1b1	Aldehyde dehydrogenase X, mitochondrial	21	46	2.248
O70400	Pdlim1	PDZ and LIM domain protein 1	8	43	2.182
Q7TMN7	Anxa4	Annexin	13	48	2.171
Q3TIU3	Ahsg	Alpha-2-HS-glycoprotein	8	35	2.162
A6ZI44	Aldoa	Fructose-bisphosphate aldolase	12	42	2.162
Q99PT1	Arhgdia	Rho GDP-dissociation inhibitor 1	7	43	2.142
F6WHQ7	Gstm1	submitted name: Glutathione S-transferase, mu 1	12	52	2.126
Q921I1	Tf	Serotransferrin	43	56	2.122
P54071	Idh2	Isocitrate dehydrogenase [NADP], mitochondrial	29	52	2.1
P18760	Cfl1	Cofilin-1	12	66	2.099
P21107-2	Tpm3	Isoform 2 of tropomyosin alpha-3 chain	21	63	2.065
Q6IRU2	Tpm4	Tropomyosin alpha-4 chain	13	56	2.014
P40124	Cap1	Adenylyl cyclase-associated protein 1	12	47	2
Q64435	Ugt1a6	UDP-glucuronosyltransferase 1–6	13	31	0.494
Q78JT3	Haao	3-hydroxyanthranilate 3,4-dioxygenase	14	57	0.49
Q9DBG1	Cyp27a1	Sterol 26-hydroxylase, mitochondrial	15	37	0.456
Q91VA0	Acsm1	Acyl-coenzyme A synthetase ACSM1, mitochondrial	21	52	0.456
G3X925	Pklr	Pyruvate kinase	28	55	0.446
O88587	Comt	Catechol O-methyltransferase	13	38	0.439
Q3V117	Acly	ATP-citrate synthase	28	32	0.439
Q9QXF8	Gnmt	Glycine N-methyltransferase	14	55	0.438
P33267	Cyp2f2	Cytochrome P450 2F2	25	54	0.369
A0A0R4J135	Selenbp2	Methanethiol oxidase	35	82	0.357
Q8R084	Ugt2b1	UDP-glucuronosyltransferase	16	37	0.348
Q91WU0	Ces1f	Carboxylesterase 1F	24	54	0.345
P19096	Fasn	Fatty acid synthase	85	45	0.344
Q99P30	Nudt7	Peroxisomal coenzyme A diphosphatase NUDT7	10	36	0.331
Q8BGL3	Sult2a8	Sulfotransferase 2A8	16	54	0.306
Q561M8	Cps1	submitted name: Cps1 protein	27	87	0.288
P19157	Gstp1	Glutathione S-transferase P 1	12	62	0.252
P40936	Inmt	Indolethylamine N-methyltransferase	12	60	0.216
Q8QZR3	Ces2a	Pyrethroid hydrolase Ces2a	15	40	0.209
Q8VCU1	Ces3b	Carboxylesterase 3B	16	37	0.179
P11589	Mup2	Major urinary protein 2	22	81	0.154
P06801	Me1	NADP-dependent malic enzyme	17	38	0.1
Q58EV3	Mup7	submitted name: major urinary protein 1	21	78	0.099
Q3TB63	Hspa8	Heat shock cognate 71 kDa protein	27	59	0.01

### Identifying Pathways Related with Progression of Fibrosis

**Fig. 3:**
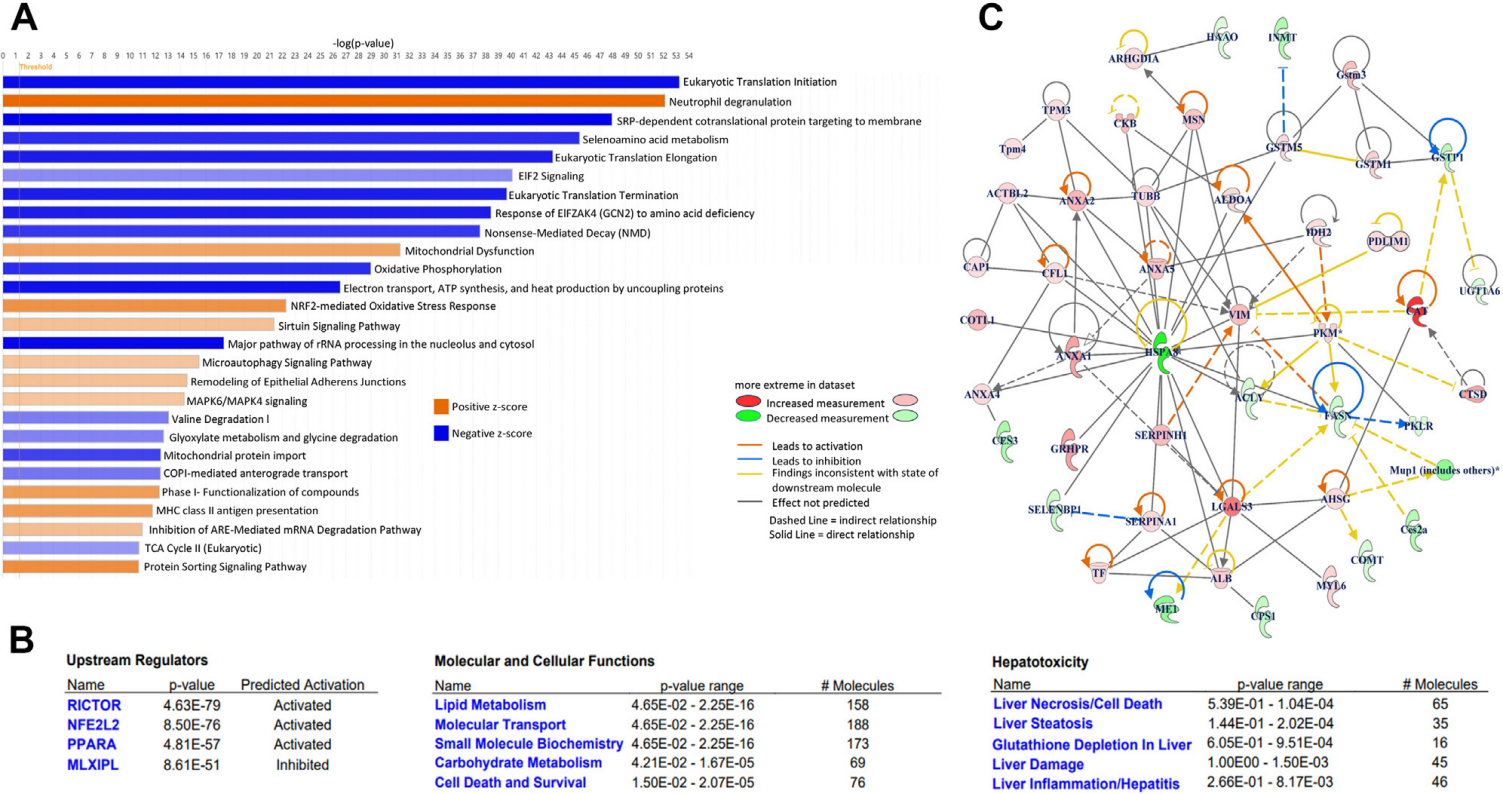
**Network analysis using ingenuity pathway analysis (IPA).***A*, canonical pathway analysis of differentially expressed proteins (n = 2651). *Blue color* indicates downregulated pathways, whereas *orange color* shows upregulated pathways. *B*, activated or inhibited upstream regulators and number of proteins identified in varied molecular and cellular functions along with hepatoxicity analysis. *C*, network interactions of the proteins found in Proteome Discoverer (version 2.5) after selection using the following criteria: Protein FDR confidence: High; Master protein; Peptide number ≥7; coverage ≥30 and abundance ratio ≤0.5 and ≥ 2.0 (Proteins with no interactions were excluded from the graph). FDR, false discovery rate.

IPA was performed to identify molecular mechanisms associated with fibrotic progression in NASH. This approach allowed us to integrate our experimental data with existing biological knowledge to elucidate the molecular mechanisms underlying fibrosis in NASH. The most enriched canonical pathways are shown in Figure 3*A*. Four distinct categories were revealed: protein production, cellular stress and defense, cellular processes and communication, and metabolism. The first category, protein production, showed downregulation in most of the related pathways, such as eukaryotic translation initiation, elongation, and termination, eukaryotic initiation factor 2 signaling, response to general control nonderepressible 2, and mitochondrial protein import. This downregulation in NASH could potentially limit the essential proteins needed for liver health and repair by decreasing protein production. The second group, cellular stress and defense, included pathways related to stress, such as neutrophil downregulation, mitochondrial dysfunction, oxidative stress, nuclear factor erythroid 2-related factor 2-mediated oxidative stress response, and nonsense-mediated decay. The increased oxidative stress response shows that the cells are attempting to cope with the increased oxidative stress caused by various factors, such as increased mitochondrial dysfunction, decreased oxidative stress, and nonsense-mediated decay. The third category, cellular processes and communication, includes pathways such as the sirtuin signaling, macroautophagy signaling, remodeling of epithelial adherens junctions, and MAPK6/MAPK4 signaling pathways. These pathways participate in signaling, protein trafficking, maintenance of cell structure, and cellular recycling. The final category included pathways such as valine metabolism and TCA cycle II, which play roles in metabolism and cellular energy production.

Subsequent upstream regulator analysis revealed that the rapamycin-insensitive companion of mammalian target of rapamycin (RICTOR) protein was the most activated upstream regulator, while MLX-interacting protein-like (MLXIPL; also known as the carbohydrate regulatory element-binding protein, ChREBP) was the most inactivated (Fig. 3*B*). MLXIPL is a key transcriptional regulator of *de novo* lipogenesis in liver.

To determine the significant biological functions and reveal transcriptional correlations among the proteins associated with NASH used in MRM scanning, 67 significantly altered proteins were subjected to network analysis (Fig. 3*C*). The network analysis which displays the protein-protein interactions among the selected proteins, reveals the complex interplay of various protein sets involved in metabolism, inflammation, oxidative stress response, cytoskeletal function, signaling, and protein maintenance. These findings support the hypothesis that these proteins are integral to the pathophysiology of NASH.

### Verification of the Biomarkers Using Targeted MS Scanning

Of a pool of 13,446 proteins, 91 were selected for MRM scan analysis of mouse liver tissues using the aforementioned filtering parameters. In contrast, 64 proteins were chosen for analysis in the mouse serum samples, which were identified as being present in the cytoplasm or cytosol, or secreted. Table 2 displays a list of these peptides, along with their fragment sequences, accession numbers, precursor ions, and product ions. Notably, information on the 27 proteins that were only scanned in mouse liver tissue can be found in Supplemental Table S2. The identified peptides were analyzed using UHPLC-LC-MS/MS for mouse tissue and serum samples, and the area was used for data comparison.

**Table 2:** List of proteins used for the multiple reaction monitoring (MRM) scanning for mouse liver and serum

Accession [Mus musculus]	Gene symbol	Peptide fragment	Modification	Precursor ion (m/z)	Product ion (m/z)	Cellular localization
Q5SWU9	*Acc1*	*FVVMVTPEDLK*	*-*	*639.35*	*702.37*	*#Cytoplasm*
Q3V117	**Acly**	SGGMSNELNNIISR	-	746.37	716.40	#Cytosol #Nucleoplasm
Q8BFZ3	**Actbl2**	GIHETTFNSIMK	-	459.90	592.31	#Cytoplasm, cytoskeleton
Q3TIU3	**Ahsg**	HAFSPVASVESASGETLHSPK	-	713.35	965.48	#Secreted
P07724	**Alb**	YMCENQATISSK	C3-C∗	716.32	1137.52	#Secreted
A6ZI44	**Aldoa**	YTPSGQSGAAASESLFISNHAY	-	1129.53	997.97	#Cytoplasm, Myofibril, sacromere
Q91Y97	*Aldob*	*ETTIQGLDGLSER*	*-*	*709.86*	*846.43*	*#Cytoplasm #Cytoskeleton*
Q4FK88	**Anxa1**	GLGTDEDTLIEILTTR	-	873.96	732.43	#Cytoplasm #cilium #Membrane #Nucleus
P07356	**Anxa2**	SALSGHLETVILGLLK	-	551.00	746.96	#Secreted #Basement membrane #Extracellular matrix
Q7TMN7	**Anxa4**	AEIDMLDIR	-	538.28	875.47	#Cytoplasmic vesicle
P48036	**Anxa5**	TPEELSAIK	-	494.27	443.75	#Cytoplasm #Dentrite #Extracellular space
Q61176	*Arg1*	*ANEELAGVVAEVQK*	*-*	*728.88*	*578.80*	#*Cytoplasm*
Q99PT1	**Arhgdia**	VAVSADPNVPNVIVTR	-	825.96	1108.65	#Cytoplasm
Q3UC32	*Arpc5*	*ALAAGGVGSIVR*	*-*	*535.82*	*744.44*	#*Cell projection #Cytoplasm #Cytoskeleton #Nucleus*
Q91YI0	*Asl*	*FVGAVDPIMEK*	*-*	*603.31*	*959.49*	#*Cytosol # Mitochondrial outer membrane*
P16460	*Ass1*	*VQVSVFK*	*-*	*403.74*	*579.38*	#*Cytoplasm*
P16015	*Ca3*	*GDNQSPIELHTK*	*-*	*446.89*	*435.54*	*#Cytoplasm*
Q8VCT4	*Ces1d*	*LDLLGNPK*	*-*	*435.26*	*415.23*	*#Cytoplasm #Endoplasmic reticulum #*Lipid droplet #Microsome
Q91WU0	**Ces1f**	EGYLHIGGTTQQAQR	-	829.91	946.47	#Cytoplasm #Endoplasmic reticulum #Lipid droplet #Microsome
P18760	**Cfl1**	LGGSAVISLEGKPL	-	670.89	1112.63	#Cytoplasm, cytoskeleton #Nucleus matrix #Cell projection
Q04447	**Ckb**	LAVEALSSLDGDLSGR	-	801.92	1006.48	#Cell membrane #Cytoplasm #Membrane #Mitochondrion
Q80X19	*Col14a1*	*VIVVITDGR*	*-*	*486.30*	*759.44*	#*Extracellular matrix #Secreted*
O88587	**Comt**	KYDVDTLDMVFLDHWK	-	675.67	845.43	#Cytosol #Cell membrane
Q9CQI6	**Cotl1**	EVVQNFAK	-	467.75	479.26	#Cytoplasm, cytoskeleton #Nucleus
P10605	*Ctsb*	EQWSNCPTIGQIR	C6-C∗	794.88	784.47	#*Cell membrane* #*Lysosome* #*Membrane #Secreted*
P12710	*Fabp1*	*GVSEIVHEGKK*	*-*	*527.78*	*449.74*	#*Cytoplasm*
P19096	**Fasn**	LLLPEDPLISGLLNSQALK	-	1017.59	847.98	#Cytoplasm
Q9QXF8	**Gnmt**	NYDYILSTGCAPPGK	C10-C∗	828.40	1258.54	#Cytoplasm
Q7M0F4	**Gst**	PMTLGYWDIR	-	626.31	1023.53	#Cytoplasm
P13745	**Gsta1**	SHGQDYLVGNR	-	623.30	1021.51	#Cytosol
Q9DCU1	*Gsta3*	*VSNLPTVK*	*-*	*429.26*	*671.41*	*#Cytoplasm*
F6WHQ7	**Gstm1**	MQLIMLCYNPDFEK	C7-C∗	901.42	1316.56	#Cytoplasm
P10649	*Gstm1*	*ADIVENQVMDTR*	*-*	*695.84*	*992.45*	#*Cytoplasm*
P15626	**Gstm2**	IQLAMVCYSPDFEK	C7-C∗	850.91	1045.43	#Cytoplasm
P19639	**Gstm3**	FNLGLDFPNLPYLIDGSHK	-	720.71	893.46	#Cytoplasm
P19157	**Gstp1**	MPPYTIVYFPVR	-	741.89	1254.69	#Cytoplasm #Nucleus #Mitochondrion
Q78JT3	**Haao**	TGKPNPDQLLK	-	404.23	529.35	#Cytoplasm, cytosol
P40936	**Inmt**	EIIVTDYTPQNLQELQK	-	1016.53	1097.59	#Cytoplasm
Q3TJX0	**Lcp1**	AECMLQQAER	C3-C∗	618.28	518.24	#Cytoplasm
P16045	*Lgals1*	*SFVLNLGK*	*-*	*439.26*	*643.41*	*#Cytoplasm* #*Extracellular matrix #Secreted*
Q3V471	**Lgals3**	IQVLVEADHFK	-	433.58	529.29	#Spliceosome #Secreted
P14152	*Mdh1*	*VIVVGNPANTNCLTASK*	*C12-C∗*	*879.46*	*1176.57*	#*Cytoplasm*
P06801	**Me1**	AIVVTDGER	-	480.26	775.39	#Cytoplasm
P26041	**Msn**	ESEAVEWQQK	-	617.29	718.35	#Cytoplasm #Cytoskeleton #Membrane
P11589	**Mup2**	IEDNGNFRLFLEQIHVLEK	-	772.08	1037.05	#Secreted
P04939	*Mup3*	*ENIIDLTNVNR*	*-*	*650.85*	*831.43*	#*Secreted*
Q58EV3	**Mup7**	IEDNGNFRLFLEQIHVLEK	-	772.08	1037.05	#Secreted
P27773	*Pdia3*	*LSKDPNIVIAK*	*-*	*399.91*	*542.82*	#*Endoplasmic reticulum*
O70400	**Pdlim1**	DFEQPLAISR	-	588.31	656.41	#Cytoplasm, cytoskeleton
Q3U7V7	*Pfn1*	*TFVSITPAEVGVLVGK*	*-*	*808.96*	*1069.63*	*#Cytoplasm #Cytoskeleton*
P52480	**Pkm**	LAPITSDPTEAAAVGAVEASFK	-	1073.05	981.50	#Cytoplasm #Nucleus
P52480–2	**Pkm**	LLFEELVR	-	509.80	792.42	#Cytoplasm #Nucleus
Q64374	*Rgn*	*VAVDAPVSSVALR*	*-*	*642.37*	*828.49*	*#Cytoplasm*
P50543	*S100a11*	*TEFLSFMNTELAAFTK*	*-*	*925.46*	*1359.66*	*#Cytoplasm* *#Nucleus*
P27005	*S100a8*	*MVTTECPQFVQNINIENLFR*	*C6-C∗*	*1227.10*	*1731.92*	#*Cell membrane* *#Cytoplasm* #*Cytoskeleton* #*Membrane #*Secreted
P31725	*S100a9*	*LIFACHEK*	*C5-C∗*	*339.85*	*396.18*	#*Cell membrane #Cytoplasm* #Cytoskeleton #Membrane #Secreted
A0A0R4J135	**Selenbp2**	VIEASEIQAK	-	544.30	875.45	#Cytoplasm #Membrane #Nucleus
P07758	**Serpina1a**	FDHPFLFIIFEEHTQSPIFLGK	-	666.35	766.89	#Secreted
Q8BGL3	**Sult2a8**	GDPTWVQSTIANER	-	787.39	1017.53	#Cytoplasm, cytosol
Q921I1	**Tf**	GYYAVAVVK	-	485.27	749.45	#Secreted
P21107–2	**Tpm3**	IQVLQQQADDAEER	-	821.90	1061.45	#Cytoplasm #Cytosol
Q6IRU2	**Tpm4**	IQLLEEELDRAQEQLATALQNLEEAEK	-	1042.21	1490.26	#Cytoplasm, cytoskeleton
P99024	**Tubb5**	ISVYYNEATGGK	-	651.32	1002.45	#Cytoplasm, cytoskeleton
P20152	**Vim**	EMEENFALEAANYQDTIGRLQDEIQNMKEEMAR	-	979.95	1046.84	#Cell membrane #Cytoplasm #Cytoskeleton

Bold: Experimental; Italic: Literature; C9-C∗: C9-Carbamidomethyl (57.02 Da).

**Fig. 4:**
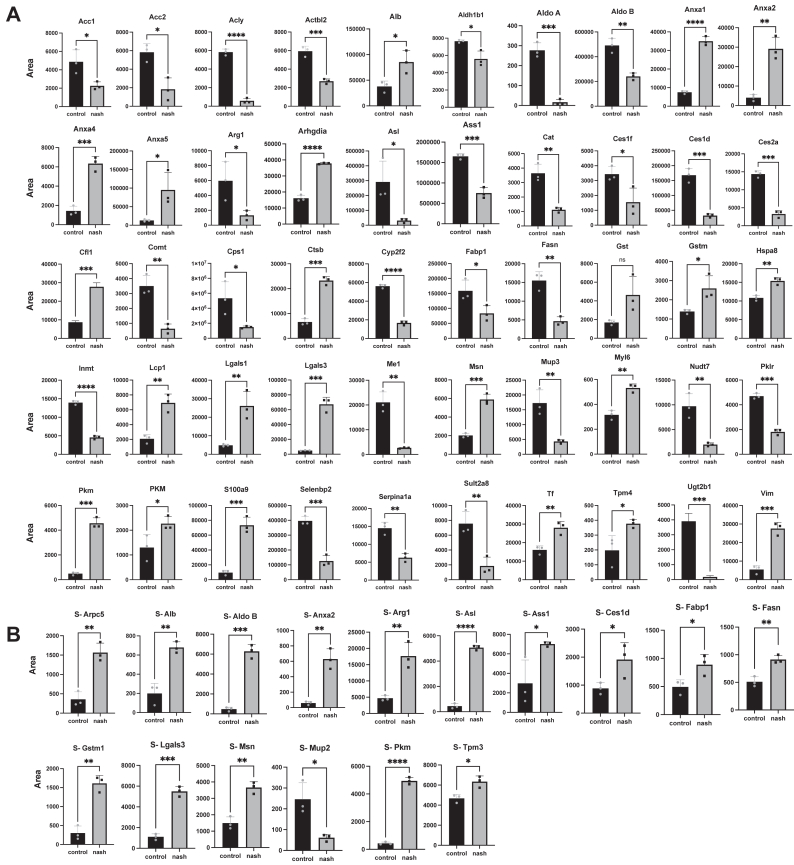
**Quantitation of the control and CDAA-diet mice fed groups by multiple reaction monitoring (MRM) for NASH biomarker candidates in liver and serum samples**. MRM scanning of mouse liver (*A* and *B*) mouse serum samples (n = 3, each group). Statistical analysis was performed using Student’s *t* test in GraphPad Prism (∗*p* < 0.05, ∗∗*p* < 0.01, and ∗∗∗*p* < 0.001). CDAA, choline-deficient L-amino acid; NASH, nonalcoholic steatohepatitis.

The MRM scan analysis of mouse serum and tissue samples revealed significant alterations in the expression levels of several proteins. Specifically, the tissue samples showed 21 increased and 29 decreased protein levels. Proteins associated with inflammation (annexin, *Anxa1*, annexin A2, *Anxa2*; galectin-3, *Lgals3*) were significantly upregulated. Moreover, enzymes involved in fatty acid metabolism (acetyl-Coa carboxylase 1, *Acc1*; acetyl-Coa carboxylase 2, *Acc2*; fatty acid synthase, *Fasn*; fatty acid binding protein, *Fabp1*), the urea cycle (arginase 1, *Arg1*; carbamoyl-phosphate synthase 1, *Cps1*; Arginosuccinate lyase, *Asl*) and detoxification enzymes (carboxylesterase 1f, *Ces1f*; carboxyesterase 1d, *Ces1d*; Cytochrome p450 2f2, *Cyp2f2*; Glutathione s-transferase, *Gst*) were downregulated (Fig. 4*A* and Table 3).

**Table 3:** Summary of significantly altered proteins in mouse liver with prolonged CDAA-HF diet

Liver			
Abbreviation	Protein	Increased/Decreased	Log2 FC (MRM)
Lgals3	Galectin-3	↑	3.75
Pkm	Isoform M1 of pyruvate kinase PKM	↑	3.23
S100a9	Protein S100-A9	↑	2.94
Anxa5	Annexin A5	↑	2.89
Anxa2	Annexin A2	↑	2.86
Lgals1	Galectin-1	↑	2.43
Vim	Vimentin	↑	2.33
Anxa	Annexin	↑	2.22
Anxa4	Annexin A4	↑	2.14
Ctsb	Cathepsin B	↑	1.82
Lcp1	Plastin-2	↑	1.74
Cfl1	Cofilin-1	↑	1.68
Msn	Moesin	↑	1.54
Arhgdia	Rho GDP-dissociation inhibitor 1	↑	1.23
Alb	Albumin	↑	1.17
Gst	Glutathione S-transferase	↑	1.04
Tpm4	Tropomyosin alpha-4 chain	↑	0.94
Pkm	Pyruvate kinase PKM	↑	0.80
Tf	Serotransferrin	↑	0.80
Myl6	submitted name: Myosin, light polypeptide 6	↑	0.75
Hspa8	Heat shock cognate 71 kDa protein	↑	0.51
Aldh1b1	Aldehyde dehydrogenase X, mitochondrial	↓	−0.45
Fabp1	Fatty acid-binding protein, liver	↓	−0.93
Gstm1	Glutathione S-transferase Mu 1	↓	−0.97
Aldob	Fructose-bisphosphate aldolase B	↓	−1.03
Acc2	Acetyl-coa carboxylase 2	↓	−1.07
Acc1	Acetyl-coa carboxylase 1	↓	−1.11
Ass1	Argininosuccinate synthase	↓	−1.13
Actbl2	Beta-actin-like protein 2	↓	−1.14
Ces1f	Carboxylesterase 1F	↓	−1.15
Serpina1a	Alpha-1-antitrypsin 1–1	↓	−1.21
Pklr	Pyruvate kinase	↓	−1.38
Arg1	Arginase-1	↓	−1.46
Inmt	Indolethylamine N-methyltransferase	↓	−1.62
Selenbp2	Methanethiol oxidase	↓	−1.66
Cat	catalase	↓	−1.70
Fasn	Fatty acid synthase	↓	−1.73
Cyp2f2	Cytochrome P450 2F2	↓	−1.75
Cps1	Carbamoyl-phosphate synthase	↓	−1.84
Mup3	Major urinary protein 3	↓	−2.00
Sult2a8	Sulfotransferase 2A8	↓	−2.02
Ces2a	Pyrethroid hydrolase Ces2a	↓	−2.14
Nudt7	Peroxisomal coenzyme A diphosphatase NUDT7	↓	−2.30
Ces1d	Carboxylesterase 1D	↓	−2.37
Comt	Catechol O-methyltransferase	↓	−2.45
Me1	NADP-dependent malic enzyme	↓	−2.99
Asl	Argininosuccinate lyase	↓	−3.21
Acly	ATP-citrate synthase	↓	−3.32
Aldoa	Fructose-bisphosphate aldolase	↓	−4.06
Ugt2b1	UDP-glucuronosyltransferase	↓	−4.45

Mouse serum MRM revealed significantly increased levels of Anxa2 and Lgals3 (Fig. 4*B* and Table 4). In contrast to the mouse liver results, the serum results also showed elevated levels of Arg1, Asl, Fasn, and Aldo B. Additionally, increased levels of actin-related 2/3 complex subunit 5 (Arpc5), albumin (Alb), arginosuccinate synthase (Ass1), moesin (Msn), pyruvate kinase PKM (Pkm), isoform 2 of the tropomyosin alpha-3 chain (Tpm3), and NADP-dependent malic enzyme (Me1) were observed. Furthermore, the scanning results revealed only one downregulated protein: major urinary protein 2 (Mup2). These changes suggest alterations in urea production, energy metabolism, and inflammatory proteins, reflecting metabolic adaptations in response to NASH-induced stress.

**Table 4:** Summary of proteins with significantly altered expression in mouse serum after prolonged CDAA-HF diet intake

Serum			
Abbreviation	Protein	Increased/Decreased	Log2 FC (MRM)
Aldo B	Fructose-bisphosphate aldolase B	↑	3.72
Pkm	Pyruvate kinase PKM	↑	3.52
Asl	Argininosuccinate lyase	↑	3.52
Anxa2	Annexin A2	↑	3.41
Gstm1	Glutathione S-transferase Mu 1	↑	2.42
Lgals3	Galectin-3	↑	2.31
arpc5	Actin-related protein 213 complex subunit 5	↑	2.13
Arg1	Arginase-1	↑	1.92
Alb	Albumin	↑	1.77
Msn	Moesin	↑	1.29
Ass1	Argininosuccinate synthase	↑	1.23
Ces1d	Carboxylesterase 1D	↑	1.12
Fabp1	Fatty acid-binding protein, liver	↑	0.85
Fasn	Fatty acid synthase	↑	0.84
Tpm3	Isoform 2 of tropomyosin alpha-3 chain	↑	0.44
Mup2	Major urinary protein 2	↓	−2.00

Abbreviations: CDAA-HF, choline-deficient, L-amino acid-defined high fat; MRM, multiple reaction monitoring.

Summary of MRM scans of mouse serum samples. The down arrow represents decreased protein expression, whereas the upper arrow shows increased protein expression.

To observe the prolonged effect of the CDAA-HF diet in mice, MRM scanning of the biomarker candidates was performed at weeks 6, 14, 16, and 18 (Supplemental Figs. S4 and S5). The results showed that the elevation of some proteins in mouse serum, such as Lgals3, Ass1, Arg1, and Aldo B, increased over time.

### Immunoblotting Assay Validation of Biomarkers

**Fig. 5:**
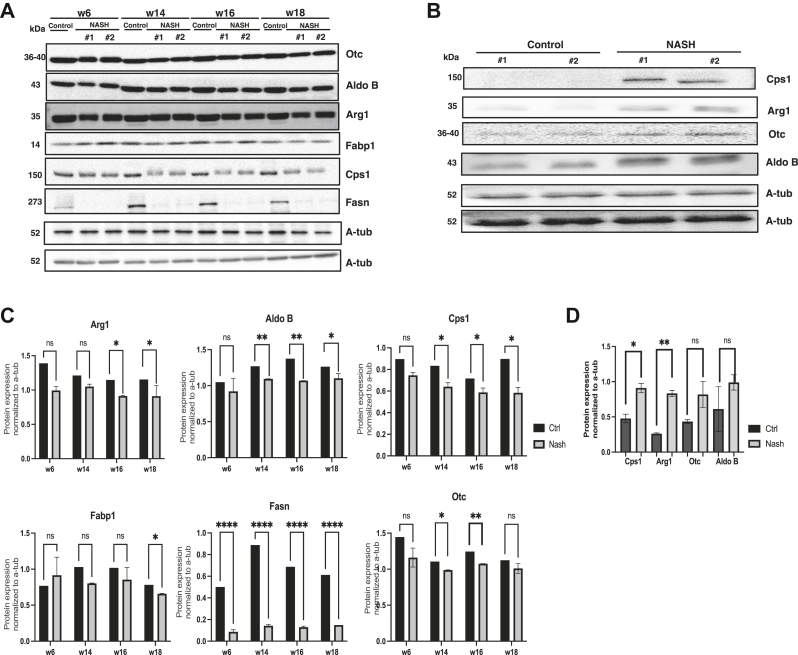
**Effects of prolonged CDAA diet intake on mouse liver and serum.** Immunoblotting and relative expression of Otc, Aldo B, Arg1, Fabp1, Cps1, and Fasn proteins in mouse liver (*A* and *B*) and serum (*C* and *D*). Statistical analysis was performed using Student’s *t* test (∗*p* < 0.05, ∗∗*p* < 0.01, ∗∗∗*p* < 0.005, and ∗∗∗∗*p* < 0.001). ALDO B, fructose-bisphosphate aldolase B; ARG1, arginase 1; CDAA, choline-deficient L-amino acid; Cps1, carbamoyl-phosphate synthase 1; Fasn, fatty acid synthase; Fabp1, fatty acid binding protein 1; Otc, ornithine transcarbamylase.

Western blotting was conducted to examine the expression levels of proteins that exhibited significant alterations in the MRM scans of liver tissues implicated in different metabolic pathways, including Otc, Aldo B, Arg1, Fasn, Cps, and Fabp1. All proteins were downregulated in the mouse liver following a prolonged CDAA-HF diet (Fig. 5, *A* and *C*). The observed downregulation of Otc, Aldo B, Arg1, Fasn, and Cps1 was consistent with previous studies reporting their involvement in various metabolic pathways, including amino acid metabolism, gluconeogenesis, and fatty acid synthesis.

Immunoblotting assays of serum samples showed a significant elevation in the urea cycle proteins Cps1 and Arg1 (Fig. 5, *B* and *D*). Otc and Aldo B also increased, but the increase was not statistically significant. Fasn and Fabp1 were not detected in serum samples.

### Verification of Candidate Biomarkers in Human Cohort

**Fig. 6:**
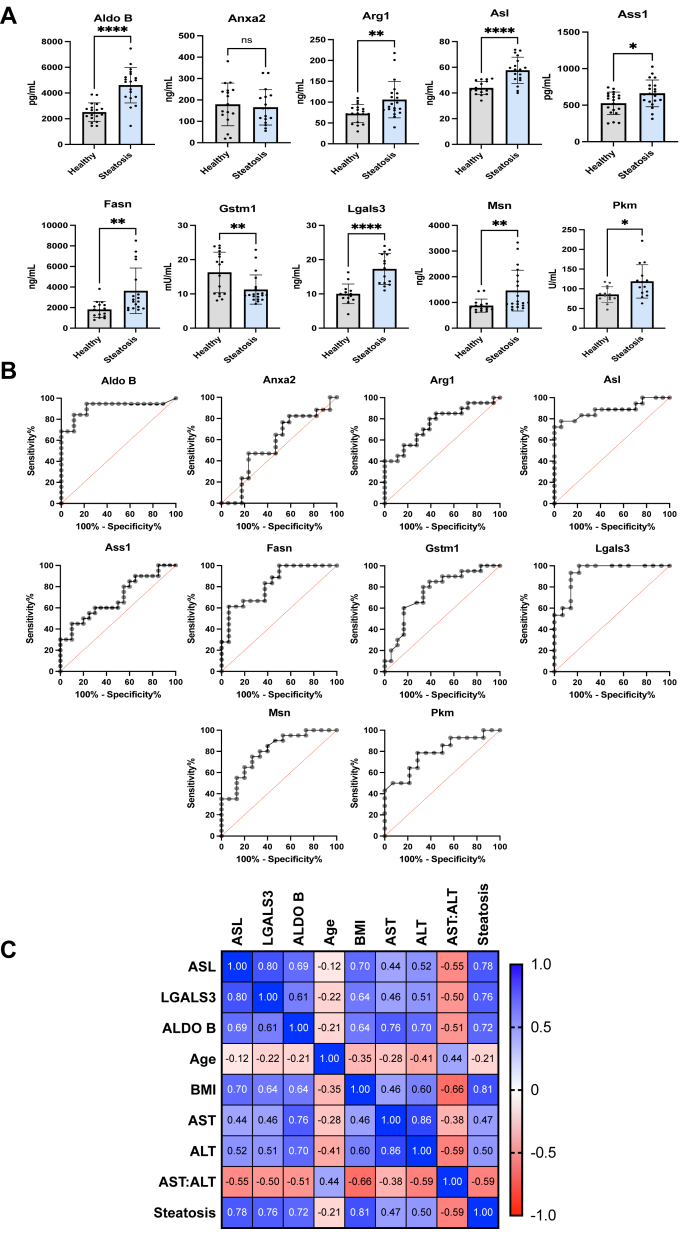
**Asl, Aldo B, and Lgals3 as potential biomarkers for early diagnosis of NASH.***A*, bar graph of potential biomarker candidates analyzed by commercially available ELISA kits. *B*, ROC curve assessment of steatosis identification from human serum. *C*, correlation matrix analysis of the selected biomarker candidates with conventional markers. Statistical analysis was performed using a one-way Student’s *t* test in GraphPad Prism (∗*p* < 0.05, ∗∗*p* < 0.01, and ∗∗∗*p* < 0.001). ALDO B, fructose-bisphosphate aldolase B; ALT, alanine aminotransferase; ANXA2, annexin 2; ARG1, arginase 1; ASL, arginosuccinate lyase; ASS1, argininosuccinate synthase 1; AST, aspartate aminotransferase; BMI, body mass index; FASN, fatty acid synthase; LGALS3, galectin-3; MSN, moesin; NASH, nonalcoholic steatohepatitis; PKM, pyruvate kinase M2; ROC, receiver operating characteristic.

To verify our findings and identify robust biomarkers for diagnosis, we conducted a final verification using ELISA with human serum samples. We selected candidate biomarkers based on mouse serum data, focusing on proteins upregulated in both tissue and serum (Anxa2 and Lgals3). Significant alterations were observed in both the liver and serum, although the specific changes differed between the two sample types (Aldo B, Fasn, Asl, Ass1, Pkm, Alb, and Msn). Notably, all the proteins, except Anxa2, exhibited consistent upregulation in human serum when compared with mouse serum, enhancing their potential as clinical biomarkers (Fig. 6*A*). We analyzed the ROC curve to demonstrate the diagnostic ability of all proteins (Fig. 6*B*). Aldo B, Arg1, Asl, Ass1, Fasn, Lgals3, Msn, and Pkm showed significance, at *p*-values <0.05. Among all the proteins, Aldo B, Asl, and Lgals3 demonstrated the best performance in detecting steatohepatitis in human serum samples. Asl showed exceptional diagnostic accuracy among all the candidates, with a *p*-value <0.001, an AUC of 0.979, and exceptional sensitivity and specificity values of 86.7 and 100.0%, respectively. Similarly, Lgals3 showed the second-highest diagnostic accuracy with a *p*-value <0.001, an AUC value 0.965, along with 100% sensitivity and 91.7% specificity. Additionally, Aldo B exhibited an AUC score of 0.917 with a *p*-value <0.001, along with 93.7% sensitivity and 80.0% specificity (Table 5).

**Table 5:** ELISA receiver operating characteristic curve analysis summary

Biomarker	AUC	Criterion	*p*-value	Sensitivity	Specificity
Anxa2	0.59	≤214.5	0.421	86.7	42.9
Ass1	0.693	>522	0.023	80	45
Arg1	0.756	>77.5	0.001	80	61.1
Pkm	0.802	>88	<0.001	80	71.4
Msn	0.806	>913.6	<0.001	76.5	75
Fasn	0.821	>1417.3	<0.001	100	53.8
Aldo B	0.917	>2865	<0.001	93.7	80
Lgals3	0.965	>11.4	<0.001	100	91.7
Asl	0.979	>51.2	<0.001	86.7	100

These three proteins, Aldo B, Asl, and Lgals3, were analyzed for their correlations with each other, and with other clinical markers (AST and ALT), age, and BMI using Pearson correlation (Fig. 6C). Strong positive correlations were observed between the candidate markers (Asl, Lgals3, and Aldo B) and the presence of steatohepatitis, with correlation coefficients of 0.78, 0.76, and 0.72, respectively. In addition to the biomarkers identified in this study, the presence of steatohepatitis significantly correlated with BMI with a correlation coefficient of 0.80. The correlation between AST and ALT levels and the presence of steatohepatitis was insufficient, as indicated by the correlation coefficients of 0.50 and 0.47, respectively. Conversely, the presence of steatohepatitis negatively correlated with the AST/ALT ratio (−0.59 correlation coefficient).

## Discussion

NASH is a severe form of NAFLD characterized by liver inflammation and cell damage, in addition to the presence of excess fat in the liver. The common feature of these two entities is the accumulation of fat in hepatocytes (>5% of total liver weight), but NASH is characterized by a more severe liver injury, including ballooning of hepatocytes, inflammatory cell infiltration, and progressive fibrosis (1). NASH can progress to advanced liver fibrosis, cirrhosis, and liver cancer and is associated with an increased risk of liver-related complications and mortality. Improving patient outcomes and creating focused treatments depend on understanding the molecular processes underlying the pathogenesis of NASH and recognizing diagnostic biomarkers.

Recently, some biomarkers have been suggested for detecting the presence or absence of NASH by combining biochemical and clinical variables, such as sex, age, the BMI, serum levels of triglycerides, the AST/ALT ratio, AST, gamma-glutamyl transferase, homeostatic model assessment of insulin resistance, and cholesterol levels (6, 13). Although the combination of these parameters could provide insights into NASH progression, they are not specific to the liver. For instance, AST and ALT levels may be elevated under various conditions, such as viral infection or hepatotoxic drug consumption (14). Modern MS technology, along with efficient methods, has enabled the identification of new markers for NAFLD and NASH by facilitating quick analysis of thousands of proteins. Some noninvasive biomarkers suggested by previous studies include apolipoprotein A1, plasma cytokeratin 18, actin, and lumican (5). However, most biomarkers have failed to show liver specificity. To investigate the proteomic differences associated with NASH and identify new early diagnostic biomarkers, a well-established dietary model involving a CDAA-HF diet in C57BL/6J mice was used. The CDAA-HF diet and C57BL/6J mouse model were selected owing to their ability to closely mimic the metabolic and histological characteristics of human NASH, including hepatic steatosis, inflammation, and fibrosis (15, 16). This approach was selected for a similar study conducted in 2020 by Veyel *et al*, where they implemented CDAA diet to induce NASH in a Wistar rat model instead of mouse and also added 1% cholesterol to their CDAA diet. Later, they conducted MS analysis using the tandem mass tags based proteomics after 12 weeks of diet treatment and transcriptomics analysis was conducted at weeks 4, 8, and 12. In their study, they also suggested biomarker candidates related to immune response, lipid metabolism, and extracellular matrix (ECM) including an increased level of Lgals3bp (17).

Differential expression analysis using Proteome Discoverer 2.5 revealed a significant number of DEPs between the liver of control and NASH mice. Sixty-seven proteins from a vast pool of 13,446 proteins emerged as potential contributors to NASH pathology. These DEPs were then categorized as either upregulated or downregulated in NASH liver when compared with those in the controls. The downregulation of proteins in NASH liver suggests that several cellular functions may be disrupted. For example, Hspa8, a molecular chaperone known to play a significant role in protein folding, is downregulated (18). This downregulation disrupts protein homeostasis, which may cause misfolded proteins to accumulate and induce cellular stress in NASH liver. Similarly, the downregulation of enzymes involved in xenobiotic metabolism, such as Cyp450 family members, indicates impaired detoxification pathways in NASH (19). This may lead to the accumulation of toxic metabolites and accelerated disease progression. Fasn, an enzyme that plays an important role in *de novo* lipogenesis by catalyzing the conversion of acetyl-CoA and malonyl-CoA to palmitate, is also downregulated. Furthermore, there is a concern regarding impaired metabolic processes in NASH due to the downregulation of carboxylesterases (Ces). These enzymes are involved in the breakdown of different molecules to remove wide range of drugs, environmental pollutants, and endogenous compounds (20). Moreover, oxidative stress, another well-recognized feature of NASH, can be caused by the downregulation of these detoxifying enzymes along with Gst (21).

Several proteins, including galectins (1 and 3), protein S100a9, and annexins (A1, A2, and A5), were upregulated in NASH liver. These proteins are involved in various cellular functions, including inflammation and fibrosis. Their upregulation suggests an activated immune response and potential contributions to scar tissue formation, both hallmarks of NASH (22, 23, 24, 25, 26, 27). Moesin, a protein involved in cell structure and movement, was also upregulated, potentially reflecting the cellular remodeling processes that occur in NASH liver (28). The upregulation in the immune response and inflammatory proteins highlights a key aspect of NASH, which is immune cell infiltration. This infiltration promotes the secretion of inflammatory cytokines (29). The stressed hepatocyte could potentially become susceptible to the cytokine-induced cell death, which could explain the observed decline in the metabolic proteins in NASH as these proteins are primarily produced by hepatocytes (30). Our IPA revealed a comprehensive profile of differentially regulated pathways in NASH mouse liver when compared with that in the controls. The identified pathways offer significant insights into the complex molecular mechanisms associated with the pathogenesis of NASH. Alterations in specific pathways in NASH tissue samples suggest potential disruptions in multiple cellular processes. The downregulation of protein synthesis pathways, such as eukaryotic initiation factor 2, indicates that the protein production mechanism is disrupted in NASH. Their downregulation may also indicate cellular disruptions, potentially contributing to cellular stress. The increase in cellular and oxidative stress observed in NASH is consistent with this discovery (31). The majority of cellular energy comes from oxidative phosphorylation, a process involving the mitochondrial ATP synthase. However, in NASH liver, the activity of pathways related to mitochondrial function, such as electron transport, ATP synthesis, and oxidative phosphorylation, decreases, indicating that the cells cannot produce as much energy. This could have significant consequences for various cellular processes and may further contribute to the prognosis of fibrosis (32). This is due to the potential induction of endoplasmic reticulum stress through the activation of unfolded protein response triggered by misfolded proteins (33).

The upregulation of specific pathways in NASH liver may indicate cellular adaptations or responses to ongoing pathological processes. In NASH liver, the upregulation of components of the pathways related to mitochondrial dysfunction is indicative of a potentially damaged mitochondrial network that may have been caused by cellular stress or damage. In addition, pathways related to apoptosis regulation, NRF2-mediated oxidative stress response, and noncanonical NF-kB signaling were upregulated, which could indicate an activated cell death program and an effort to manage oxidative stress (34). Chronic activation of the hepatocyte NF-kB signaling pathway is one of the causes of *de novo* lipogenesis and cholesterol synthesis (35). These findings support the notion that the NASH liver undergoes cellular stress and damage.

The data-dependent acquisition (DDA) is a discovery-based approach that offers a broad overview of the proteome but may lack sensitivity for low-abundance proteins. The DDA results which are illustrated in Supplemental Fig. S6. In this study, MRM was selected for verification due to its enhanced sensitivity and specificity in detecting these proteins, which is particularly important in the complex biological matrix of liver tissue whereas. All candidate proteins were selected using DEPs and literature review and the peptide fragments were selected using PD 2.5 software based on their uniqueness to the corresponding protein and their fragmentation pattern and abundances, as mentioned earlier. In total, 91 proteins, including 67 DEPs and 24 from the literature (6, 16, 21, 36, 37, 38, 39), were selected for mouse liver analysis for targeted MS analysis. By focusing on a defined set of proteins, we aimed to provide more reliable quantification that supports our findings regarding disease mechanisms. Notably, some discrepancies were observed between DDA and MRM results, which may be raised due to the sensitivity differences of the methods, instruments, and/or fragmentation pattern differences.

Notably, a cluster of proteins associated with inflammatory and fibrogenic processes, including Lgals3, Pkm, and S100a9, was significantly upregulated, with fold changes of 3.75, 3.23, and 2.94, respectively. In contrast, a subset of proteins involved in key metabolic pathways was downregulated. These included enzymes critical for fatty acid metabolism (Acc1, *-1.11*; Fasn,*-1.73*; Fabp1, *-0.93*), urea cycle (Cps1*, -1.84*; Ass1, -*1.13*), and detoxification processes (Ces1f, *-1.15*; Ces1d, *-2.37;* Cyp2f2, *-1.75*). This suggests the potential disruption of lipogenesis, nitrogen metabolism, and cellular defense mechanisms in response to oxidative stress.

The validation of the MRM scan of the liver tissue was confirmed through the analysis of mouse serum samples by scanning proteins located in the cytoplasm, cytosol, or secreted compartments. Among the proteins analyzed, 16 showed significant changes; of these, 15 were upregulated and one downregulated. The elevated levels of Anxa2, Msn, and Lgals3 were consistent with the liver tissue results. Anxa2 is a membrane-bound Ca^2+^-dependent protein that plays a role in a wide range of biological activities, such as cell proliferation, cell death, angiogenesis, and metastasis. In a study using the high fat diet mouse model of NAFLD over 16 weeks, it was proposed that Anxa2 may contribute to lipid buildup and liver damage by inhibiting the autophagy flux controlled by AMPK-mTOR (40). Increased Anxa2 correlates with p-STAT3 activation, which promotes caspase-1-mediated hepatocyte pyroptosis and fibrosis in NASH (24). As mentioned earlier, Msn regulates various cellular functions, including cell adhesion, polarity, and migration. It is suggested to be involved in hepatic fibrosis through the binding of integrins and ECM ligands and focal adhesion proteins, which further activates several actins and ultimately allows nuclear translocation of myocardin-related transcription factor A. The interaction between myocardin-related transcription factor A and serum-response factor with regulatory elements is crucial in controlling the differentiation of myofibroblasts and the synthesis of ECM; it also plays an important role in cytoskeletal function in hematopoietic stem cells, potentially leading to fibrogenesis (41).

Conversely, many proteins showed a decrease in the liver tissue and a significant elevation in mouse serum sample scans. For instance, Asl, one of the protein with most elevated expression in mouse serum scan (fold change *3.52*), was downregulated in tissue scans (fold change *-3.21*).

To confirm our findings and identify reliable biomarkers for diagnosis, we performed a final validation by subjecting human serum samples to ELISA. The proteins with reduced expression in tissue samples but increased expression in serum samples and those with elevated expression in both the liver and serum samples in MRM scanning were chosen for further validation. Proteins selected for the study included Anxa2, Msn, and Lgals3, which were found to be upregulated in both mouse liver tissue and serum, and Aldo B, Fasn, Asl, Ass1, and Pkm, which were significantly differentially altered in human serum when compared with the liver (ms2 fragmentation of these selected biomarkers are shown in Supplemental Fig. S3 for both serum and liver). Human serum sample analysis of all proteins, except Anxa2, showed the same elevation as in mouse serum. This discrepancy regarding Anxa2 may require further investigation. Nevertheless, the MRM scanning performed by scanning mouse serum (Supplemental Fig. S5) showed that Anxa2 levels increased in the early stages of the disease (weeks 6 and 14). However, the protein expression levels began to decrease at week 16. The fluctuation in Anxa2 levels during disease progression could explain why Anxa2 showed no significance. Additionally, the difference could have increased due to the species-specific regulations or variations in humans and mice. However, the overall consistency across species strengthens their potential as clinical biomarkers for human disease diagnosis. Among the potential candidates, the highest sensitivity and specificity values were observed with Lgals3, Aldo B, and Asl, along with high AUC values. These findings suggest that these proteins are the most promising biomarker candidates for NASH diagnosis.

Some of the identified markers have been mentioned in previous studies. For example, Asl has been suggested as a liver specific biomarker for hepatopathies. Feng *et al*. analyzed human serum samples and showed that Asl levels increased in patients with hepatopathy and remained relatively constant in patients with other diseases (42). While their study focused only on the Asl protein, our study showed increased levels of other urea cycle-related enzymes, such as Arg1 and Ass1. Lgals3 was explored in another study that compared human serum protein levels in a larger number of healthy and NASH patients; the results showed that the levels of Lgals3 were increased, along with those of other markers, such as caspase recruitment domain and interleukin 18, with 61% accuracy (43). Another study examined the plasma profiles of patients with or without cirrhosis and NAFLD and found a 341% increase in Aldo B levels in patients with NAFLD when compared with that in healthy controls (13). They also found that the levels of Lgals3-binding protein increased by 102% in NAFLD. Thus, in another study conducted in alcoholic liver disease patients’ liver and serum samples showed results consistent with our findings of Aldo B. As fibrosis progressed, Aldo B levels drop in the liver, while they initially increase in serum due to cell damage but later decrease in advanced fibrosis, indicating reduced liver function (44). Additionally, serum Fasn levels increased in patients with hepatitis; moreover, Fasn’s potential to predict liver steatosis in patients with hepatitis C virus has been suggested, as Fasn levels are related to the degree of steatosis (45). Our study validated previously suggested biomarkers, in addition to introducing other potential markers found in human serum, such as Pkm and Msn. Pkm and Msn levels increased in steatosis; however, no studies have been conducted using human serum.

Our study had some limitations. First of all, we used a small number of mice and human serum samples. Although our model closely mimicked the key features of human NASH, such as liver fat accumulation, inflammation, and fibrosis, it may not fully represent the complexity of human NASH owing to the inherent differences between animal models and human diseases. Secondly, the heterogeneous group of mice, which includes NAFLD and NASH phenotypes, could introduce variability and complicate the interpretation and precision of the results. Furthermore, proteomic analysis methods have their own set of limitations that might lead to missed or incorrect identification of some proteins. We would also like to point out that we performed bulk tissue proteomics for liver tissues to provide an average protein expression profile across all cell types present in the tissue. Although this approach is informative, it has the disadvantage of obscuring the specific changes occurring within individual cell populations.

Despite these limitations, our IPA identified a comprehensive set of differentially regulated pathways in NASH liver tissues. These findings provide valuable insights into the complex interplay between the various cellular processes that are disrupted during NASH development and progression. The molecular mechanisms underlying NASH can be better understood by focusing on these pathways and proteins involved. This information can be used to develop novel therapeutic strategies that target specific pathways or proteins to improve disease management and potentially reverse or slow the progression of NASH. In this study, we identified Aldo B, Asl, and Lgals3 as potential biomarker candidates, all of which showed high specificity (≥80.0%), sensitivity (≥86.7%), and AUC (>0.9) scores in validation studies. They could potentially provide insights into NASH development and diagnosis in clinical situations. In future studies, the suggested biomarker candidates can be validated for their use in clinical diagnosis. Overall, an in-depth screening and multilevel verification of the samples used in this study showed that lipid accumulation in liver resulted in an alteration in the mouse and human liver and serum samples. By using a multilevel verification technique, we have been able to cross-validate our results obtained from different methodologies, which strengthens the reliability of our conclusions and provides a comprehensive understanding of the protein dynamics involved in NASH. The candidate biomarkers suggested in the study might have potential clinical applications in the future. The molecular mechanism involved in the urea cycle can further be explored to elucidate the mechanisms underlying the role of these proteins in NASH development.

## Data Availability

The mass spectrometry proteomics data have been deposited to the ProteomeXchange Consortium (http://proteomecentral.proteomexchange.org) *via* the PRIDE partner repository with the dataset identifier PXD053187 and 10.6019/PXD053187. Access to the dataset can be obtained using the following credentials: username reviewer_pxd053187@ebi.ac.uk with the following password ylgUISvpupUg. Additionally, the MRM data is deposited to PASSEL database (ftp://PASS05866: CC4954ewr@ftp.peptideatlas.org/) under the identifier PASS05866, with the corresponding password CC4954ewr and PRIDE repository accessible with the username reviewer_pxd059900@ebi.ac.uk and the password t2EUZgxttbR9.

## Ethics Approval and Consent to Participate

The mice were maintained in accordance with the Institutional Animal Care and Use Committee (IACUC) standards, as followed by the Korea Institute of Science and Technology, South Korea. The animal study was granted on 2022 with the protocol number KIST-IACUC-2022 to 027. Human serum samples were obtained from the Biobank of Ajou University Hospital, a member of the Korea Biobank Network. Informed consent was obtained from all subjects involved in the study. This study was approved by the Korea Institute of Science and Technology (Approval IRB number: KIST-202312-BR-003).

## Consent for Publication

All the authors have approved the manuscript for submission.

## Supplemental data

This article contains supplemental data.

## Conflict of interest

The authors declare no competing interests.
