# Supplementary material for: Comparative Analysis of the Total Proteome in Nonalcoholic Steatohepatitis: Identification of Potential Biomarkers
Source: Mol Cell Proteomics. 2025 Jan 31;24(3):100921. doi: 10.1016/j.mcpro.2025.100921 (PMC11910689; doi:10.1016/j.mcpro.2025.100921)
Supplement: Supplementary Figure 2 [file mmc2.pdf]

COMPARATIVE ANALYSIS OF THE TOTAL PROTEOME IN NONALCOHOLIC STEATOHEPATITIS: IDENTIFICATION OF POTENTIAL BIOMARKERS

Eda Ates[1,2], Hien Thi My Ong[1,2], Seung-Min Yu[1,3], Ji-Hoon Kim[1], Min-Jung Kang[1,2,*]

[1]Center for Advanced Biomolecular Recognition, Biomedical Research Institute, Korea Institute of Science and Technology, Seoul 02792, Republic of Korea.

[2]Division of Bio-Medical Science & Technology, KIST School, University of Science and Technology, Seoul 02792, Republic of Korea.

[3]Collage of Medicine, Seoul National University, Seoul 03080, Republic of Korea.

Exploring Proteomic Changes in NASH for Biomarkers

*Corresponding author

Min-Jung Kang, Ph.D.

Principal Research Scientist

Center for Advanced Biomolecular Recognition

Korea Institute of Science and Technology

Hwarangno 14-gil 5, Seongbuk-gu, Seoul 136-791, Republic of Korea

Tel: +82-2-958-5088 Fax: +82-2-958-5059

E-mail: mjkang1@kist.re.kr

Figure S-2. Heatmap with the Heat shock cognate 71 kDa protein (Hspa8)

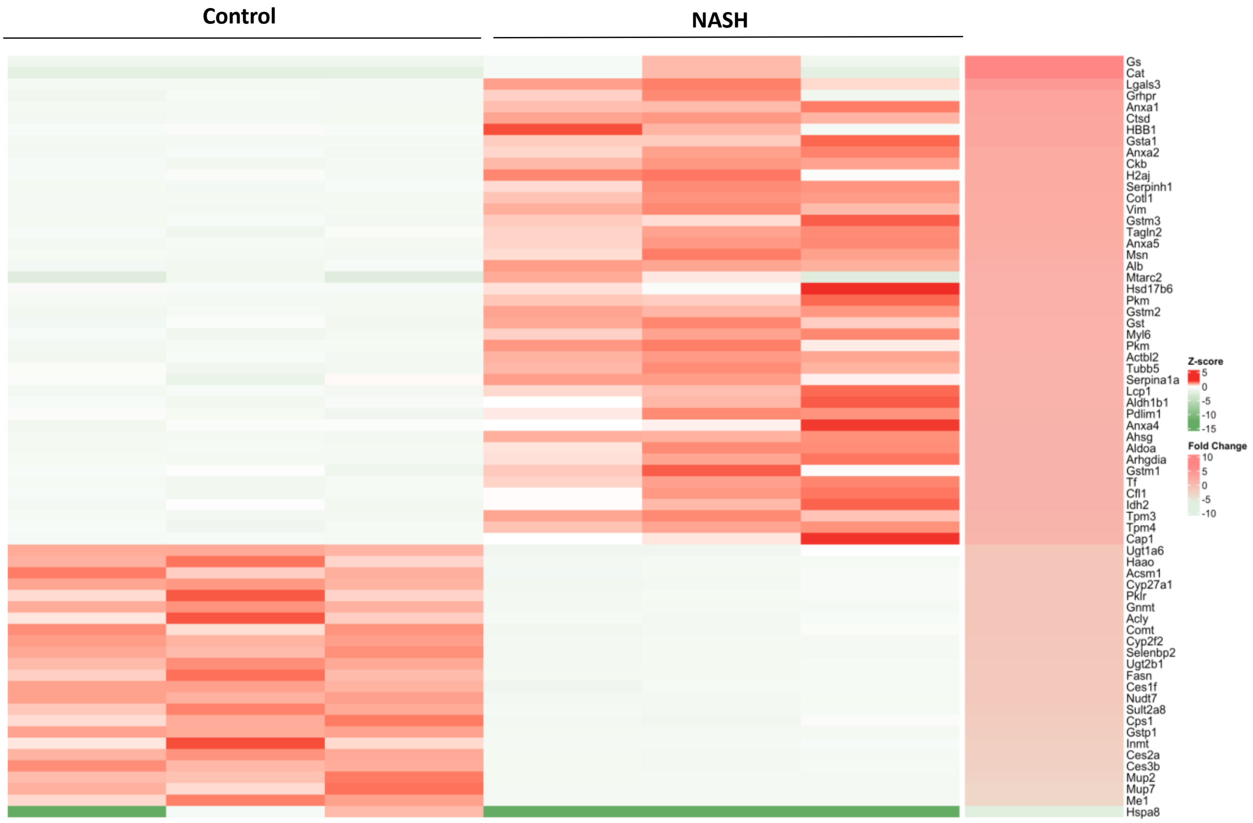

The heatmap visualisation of the differentially expressed proteins in mouse liver with 6-week of choline-deficient L-amino acid-defined high fat diet (CDAA-HF).
